# Targeting chemoresistance and mitochondria-dependent metabolic reprogramming in acute myeloid leukemia

**DOI:** 10.3389/fonc.2023.1244280

**Published:** 2023-09-07

**Authors:** Lili Feng, Philip Y. Zhang, Wenda Gao, Jinming Yu, Simon C. Robson

**Affiliations:** ^1^ Shandong Provincial Key Laboratory of Radiation Oncology, Cancer Research Center, Shandong Cancer Hospital and Institute, Shandong First Medical University and Shandong Academy of Medical Sciences, Jinan, China; ^2^ Center for Inflammation Research, Department of Anesthesia, Critical Care & Pain Medicine, Beth Israel Deaconess Medical Center, Harvard Medical School, Boston, MA, United States; ^3^ Antagen Institute for Biomedical Research, Canton, MA, United States; ^4^ Department of Radiation Oncology, Shandong Cancer Hospital and Institute, Shandong First Medical University and Shandong Academy of Medical Sciences, Jinan, China; ^5^ Department of Medicine, Division of Gastroenterology/Hepatology, Beth Israel Deaconess Medical Center, Harvard Medical School, Boston, MA, United States

**Keywords:** acute myeloid leukemia, chemoresistance, mitochondrial metabolism, mitotherapy, metabolic reprogramming

## Abstract

Chemoresistance often complicates the management of cancer, as noted in the instance of acute myeloid leukemia (AML). Mitochondrial function is considered important for the viability of AML blasts and appears to also modulate chemoresistance. As mitochondrial metabolism is aberrant in AML, any distinct pathways could be directly targeted to impact both cell viability and chemoresistance. Therefore, identifying and targeting those precise rogue elements of mitochondrial metabolism could be a valid therapeutic strategy in leukemia. Here, we review the evidence for abnormalities in mitochondria metabolic processes in AML cells, that likely impact chemoresistance. We further address several therapeutic approaches targeting isocitrate dehydrogenase 2 (IDH2), CD39, nicotinamide phosphoribosyl transferase (NAMPT), electron transport chain (ETC) complex in AML and also consider the roles of mesenchymal stromal cells. We propose the term “mitotherapy” to collectively refer to such regimens that attempt to override mitochondria-mediated metabolic reprogramming, as used by cancer cells. Mounting evidence suggests that mitotherapy could provide a complementary strategy to overcome chemoresistance in liquid cancers, as well as in solid tumors.

## 1 Introduction

Acute myeloid leukemia (AML), driven by uncontrolled propagation of clones of myeloid origin, comprises a group of aggressive hematologic malignancies with characteristic genetic or epigenetic features and distinct clinical presentations. Since 2017, the U.S. Food and Drug Administration has approved eleven new drugs or combinatory regimens, and AML’s 5-year overall survival (OS) rate has been greatly improved (1–11). However, cure rates differ immensely between various age groups and are impacted by advanced stages of the disease. While 95% cure rate is expected in younger patients with non-high-risk acute promyelocytic leukemia, this is essentially incurable for older adults with refractory or resistant disease, such as in the case of complex karyotypes (12, 13). Chemotherapy alone, and in some situations, in combination with hematopoietic stem cell transplantation, remains the mainstay of AML treatment. However, nonresponse rates could be as high as 20-40% in AML patients (14). Even though the bulk of AML cells are purged upon initial treatments, therapy-resistant cells, especially leukemic stem cells (LSCs), persist and drive relapse. Relapsed/refractory (R/R) AML continues to challenge the medical community, as the 5-year survival rate here is at a bleak 10% (15). Treatment failure and relapse in AML patients have prompted continuous search for additional and synergistic therapeutic avenues to overcome chemoresistance.

Recent years have seen the development of some targeted therapies, thanks to the delineation of abnormalities in the molecular pathways leading to AML. For example, novel therapeutic options are being tested in AML using inhibitors of Fms-Like Tyrosine kinase-3 (FLT3), isocitrate dehydrogenase (IDH), B-cell lymphoma 2 (BCL-2), FOXO3A (a forkhead transcription factor) inhibitor, or with stabilizers of p53 (3–5, 16–20). However, these small molecules, as monotherapies, demonstrate only moderate anti-leukemic activities. Moreover, the efficacy of these anti-leukemia regimens is limited by the presence of specific gene mutations and the clonal heterogeneity of AML cells; especially in R/R AML (21, 22).

Increasingly, multiple evolving data have shown that the altered bone marrow (BM) niche in AML patients plays a significant role in disease ontogeny; but also provides opportunities for efficient anti-leukemia immunotherapies (23). AML immunotherapies include cancer vaccines, adoptive cell-based therapies and antibody-based biologics, such as immune checkpoint inhibitors (24–26). However, these immunotherapies await further fine-tuning in immuno-phenotyping AML cells, particularly LSCs, to decrease unwanted on-target-off-leukemia toxicity and escape of minor clones that drive relapse (27). In addition, the complicated immunological networks among immune, stroma and leukemic cells within the AML niche, appear to limit therapeutic impact of most employed immunotherapies (28).

In the BM microenvironment, AML cells are metabolically reprogrammed and may use mitochondrial oxidative phosphorylation (OXPHOS) as important energy sources (29). In addition, mounting evidence suggests that rewiring of energy metabolism is an important factor underpinning chemoresistance in AML (30, 31). The mitochondria-mediated aberrant cellular processes used by AML have led to studies on therapies modulating basic (patho)physiological processes, such as cellular metabolism, nutrient sensing and mitochondrial functionality (32).

Besides intrinsic cellular metabolic factors, intercellular communications, such as transfer of mitochondria from mesenchymal stromal cells (MSCs) to AML cells, have also been shown to be vital for development of chemoresistance (33). To elaborate the role of dysregulated mitochondria metabolism in the chemoresistance of AML and to explore the potential therapeutic targets, we review the aberrant intrinsic mitochondria metabolic processes and mitochondria trafficking from MSCs to AML as emerging targets to overcome chemoresistance in AML.

Here, we coin the word “mitotherapy” to collectively refer regimens that attempt to override the mitochondria mediated aberrant cellular processes used by AML cells.

## 2 Mitochondrial metabolic reprogramming in AML

Metabolic reprogramming in AML cells defines an area of research more than the conventional “Warburg effect” and reflects the unique dualistic requirements for mitochondria to participate in both generation of the cellular energy currency (adenosine triphosphate, ATP) and *de novo* nucleotide biosynthesis by “metabolons”. These “metabolons” are alternatively termed “purinosomes’ and comprise of dynamic bodies associated with mitochondria, containing enzymes underpinning purine biosynthesis (34). These arrangements and proximity of the purinosome to mitochondria may help facilitate channeling of intermediates from the tricarboxylic acid (TCA) cycle (aka Krebs cycle) into the pathways of purine biosynthesis required for *de novo* nucleotide generation necessary for AML blast expansion.

Established links between the purine biosynthesis pathway and mitochondrial metabolism are important for coordinating energy metabolism and nucleotide synthesis within the cells. The purine biosynthesis pathway requires high-energy inputs, which exist in the form of ATP. ATP is synthesized mainly in mitochondria, largely through OXPHOS. Moreover, several enzymes involved in purine biosynthesis are associated with, or localized within mitochondria, such as phosphoribosyl pyrophosphate amidotransferase and adenylosuccinate lyase (35). Purine biosynthesis also generates important metabolic intermediates, such as 5-phosphoribosyl-1-pyrophosphate and adenosine monophosphate (AMP), which enter the mitochondrial matrix to participate in various metabolic pathways (36). The mitochondrial electron transport chain (ETC) plays an important role in maintaining cellular redox balance (37). The purine biosynthesis pathway furthermore involves the utilization of various reducing equivalents, such as nicotinamide adenine dinucleotide phosphate (NADPH), which is generated during the pentose phosphate pathway through the malate-aspartate shuttle linked to mitochondrial structures. In essence, the mitochondrial ETC indirectly supports the redox balance that is clearly necessary for efficient purine biosynthesis.

**Figure 1:**
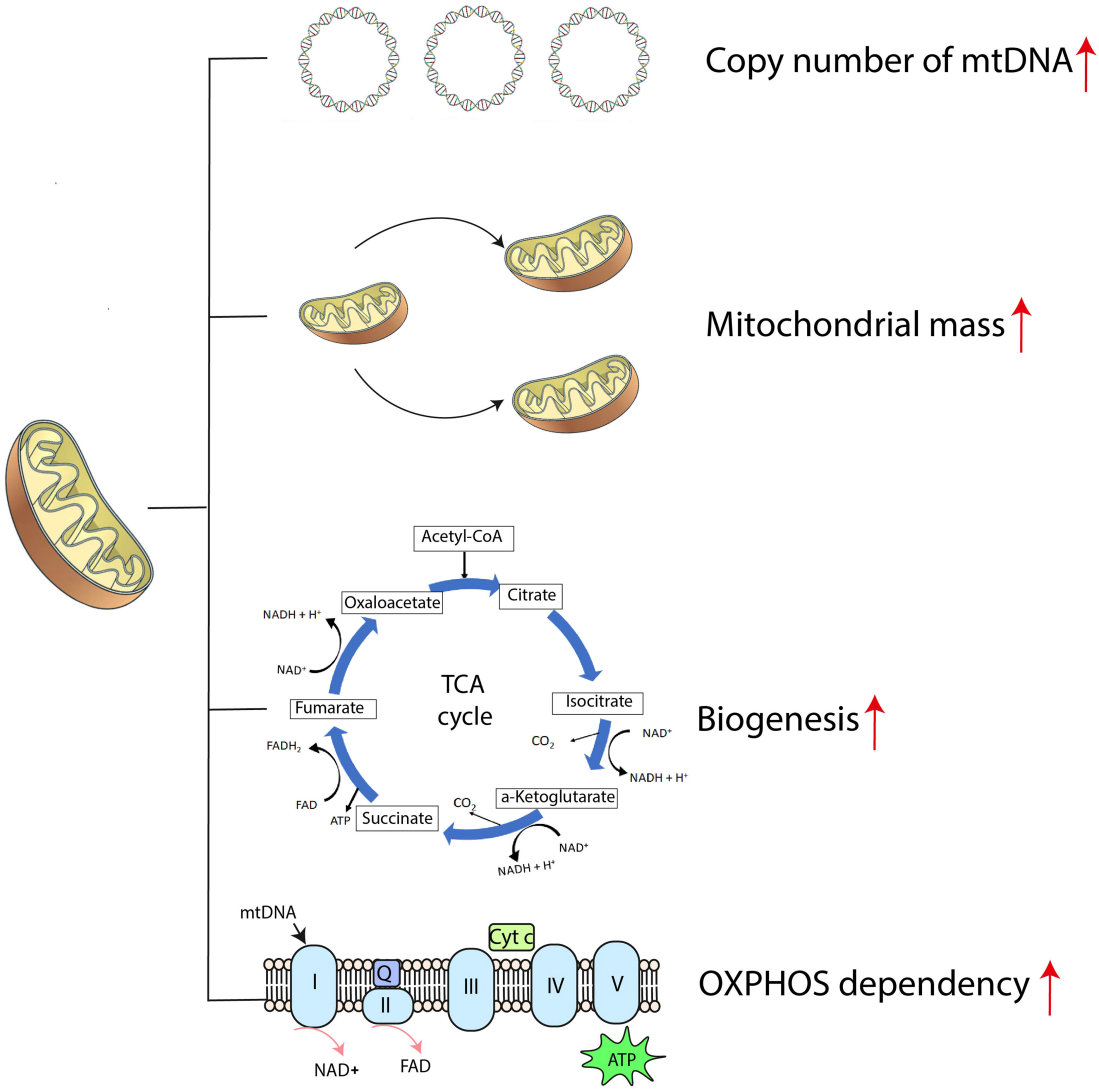
The mitochondrial bioenergetic signature of AML cells. ATP, adenosine triphosphate; Cyt c, cytochrome c; mtDNA, mitochondrial DNA; NAD^+^, nicotinamide adenine dinucleotide; OXPHOS, oxidative phosphorylation; Q, coenzyme Q; TCA cycle, tricarboxylic acid cycle.

When compared with normal hematopoietic progenitors, AML cells exhibit unique mitochondrial signatures, including higher copy numbers of mitochondrial DNA (mtDNA), greater mitochondrial mass, paradoxical dependency on OXPHOS, increased mitochondrial biogenesis and altered translation systems required for survival (**Figure 1**) (38, 39). Relapsed AML cells show enriched mitochondrial ribosomal proteins and subunits of the respiratory chain complex when analyzed with mass spectrometry, indicative of reprogrammed energy metabolism (40). All these features cause metabolic vulnerabilities in AML cells and should make these cells more susceptible to agents targeting mitochondrial function.

Mitochondria transfer is another important mechanism underpinning the higher mitochondrial mass in AML. In a co-culture system mimicking the BM niche, cultured AML cells increase their mitochondrial mass up to 14% by transfer of functional mitochondria from BM stromal cells (41). As a result, AML cells receiving the transferred mitochondria are less prone to chemotherapy-induced mitochondrial depolarization, displaying higher survival rates (41, 42).

Several proteins involved in mitochondrial calcium signaling are aberrantly expressed and located on the surface of AML cells and LSCs; these include oxysterol-binding protein (OSBP)-related proteins (ORPs), transient receptor potential melastatin 2 (TRPM2), and neurokinin-1 receptor (NK-1R). The increased expression of OSBP-related protein 4 L (ORP4L) in AML cells favors the synthesis of inositol-1,4,5-trisphosphate (IP3). This mediator activates IP3 receptors, leading to Ca^2+^ release from endoplasmic reticulum (ER) and enhanced mitochondrial respiration, which is crucial for LSCs survival. LYZ-81, an inhibitor of ORP4L, is designed to selectively eradicate LCSs *in vitro* and *in vivo* (43). TRPM2 is a Ca^2+^-operable, nonselective cation channel in response to reactive oxygen species (ROS) (44). High TRPM2 expression is positively correlated with AML proliferation and survival advantage through modulation of mitochondrial function, ROS production, and autophagy (45). NK-1R is a high-affinity receptor for substance P, which has been shown to be a growth driver in many cancers, including AML. Blocking NK-1R has been shown to induce apoptosis of AML cells *in vitro* and *in vivo* (46).

AML blasts are also characterized by ROS overproduction, which can harm redox-sensitive signaling proteins by cysteine oxidation (47). Studies on the driving factors of mitochondrial ROS overproduction in AML cells have identified some mutations in key molecules. For instance, *FLT3-internal tandem duplication (FLT3-ITD)* is a genomic marker linked with poor clinical prognosis. *FLT3-ITD*-caused ROS overproduction drives defective DNA damage repair, leading to high mutation burden and the appearance of new leukemic clones (48). Mutations in *IDH1* and *IDH2* genes result in the production of oncometabolite (R)-2-hydroxyglutarate ((R)-2-HG), exacerbating accumulation of ROS (49). In turn, ROS stimulates the proliferation of AML cells *via* an extracellular signal-regulated kinase (ERK)-dependent pathway and phosphorylation of nuclear factor kB (NF-kB) (50). However, to safeguard the self-renewal capacity, LSCs are special in low levels of ROS, which apparently results from a combination of low mitochondrial activity and high ROS-removing capacity, e.g., through autophagy pathways (51).

In a comparable manner to the role of cancer stem cells in mediating drug-resistance, distant metastasis and tumor recurrence (52), increasing evidence points to a crucial role of LSCs in AML evolution and chemoresistance, but targeting these cells remains challenging because of the difficulty in finding reliable phenotype markers. Moreover, the quiescent state of LSCs render these refractory to conventional AML therapies (53). It has been found that LSCs have unique mitochondrial metabolic signatures; these are different from normal hematopoietic stem cells (HSCs) that rely more heavily on glycolysis, LSCs demonstrate higher dependence on OXPHOS over glycolysis for the cellular energy demand (54). Residual LSCs specifically relying on mitochondrial OXPHOS are thought to be the major perpetrators responsible for AML propagation after chemotherapy (55, 56). These findings have raised possibilities for potential therapeutic interventions targeting mitochondrial metabolism to overcome LSC-mediated chemotherapy resistance.

## 3 Aberrant intrinsic mitochondria metabolic processes as emerging targets to overcome chemoresistance in AML

**Figure 2:**
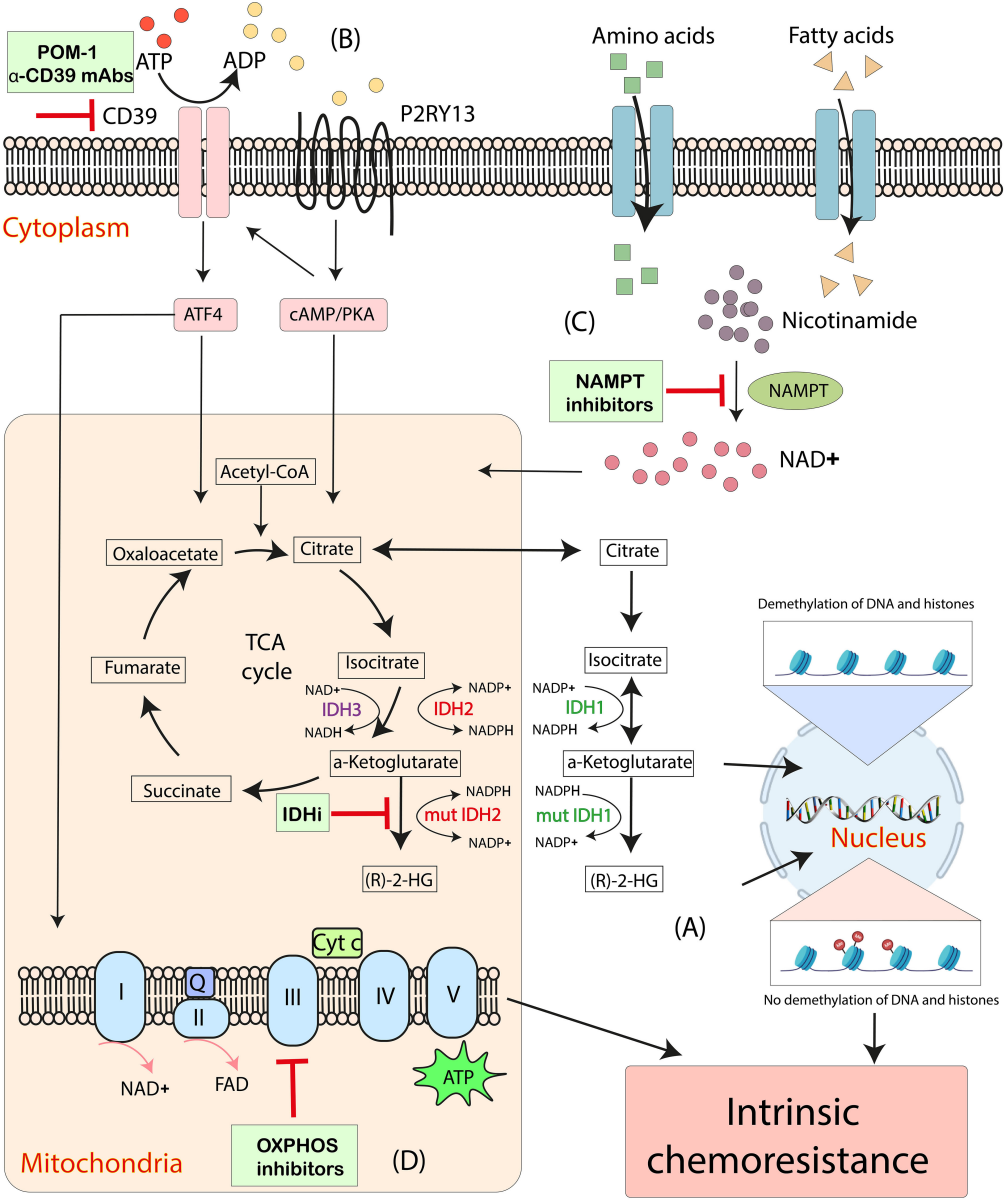
Emerging mitochondrial-targeted treatments or “mitotherapies” directed at aberrant intrinsic mitochondria metabolic processes in AML. **(A)** The accumulation of intracellular (R)-2-HG, catalyzed by mutant IDH1 and IDH2, increases DNA and histone methylation, associating with chemoresistance in AML. **(B)** CD39 promotes mitochondrial biogenesis and drives OXPHOS *via* ATF4 and a P2RY13-cAMP/PKA signaling, resulting in the intrinsic chemoresistance in AML cells. **(C)** NAD^+^ drives OXPHOS by activating the NAD^+^-dependent catabolism of amino acids and fatty acids. NAMPT inhibitors decrease mitochondrial activity and increases apoptosis in AML cells. **(D)** Inhibitions of OXPHOS cause altered oxygen consumption rates and decreased ATP production and have synergistic effects with anti-leukemia therapeutics. ADP, adenosine diphosphate; ATP, adenosine triphosphate; Cyt c, cytochrome c; IDH, isocitrate dehydrogenase; IDHi, inhibitors to IDH-mutant proteins; mut, mutant; NAD^+^, nicotinamide adenine dinucleotide; NAMPT, nicotinamide phosphoribosyl transferase; OXPHOS, oxidative phosphorylation; Q, coenzyme Q; (R)-2-HG, (R)-2-hydroxyglutarate; TCA cycle, tricarboxylic acid cycle.

Given the metabolic reprogramming in AML and the specific reliance on OXPHOS in LSCs, targeting the related pathophysiological processes provides the potential possibilities to overcome chemoresistance in AML. Here, we review those strategies targeting IDH2, CD39, nicotinamide phosphoribosyl transferase (NAMPT) and OXPHOS are putative avenues to regulate the intrinsic mitochondrial-mediated cellular processes hijacked by AML cells (**Figure 2**).

### 3.1 IDH2

Therapies targeting *IDH2* mutations serve as possible mitotherapy candidates in AML. IDH1 and IDH2 enzymes are nicotinamide adenine dinucleotide phosphate (NADP^+^-)-dependent and oxidatively catalyze isocitrate decarboxylation into α-ketoglutarate (α-KG) in TCA cycle. In AML, mutations in *IDH1* and *IDH2* occur in about 5%-10% and 10%-15% of patients, respectively (57, 58). Common early driver mutations include certain somatic changes at crucial arginine residues in the cytoplasmic enzyme IDH1 and its mitochondrial counterpart IDH2. These mutations cause novel gain-of-function in these enzymes, catalyzing α-KG to (R)-2-HG conversion (59). The accumulation of oncometabolite (R)-2-HG poisons ten-eleven translocation (TET) family enzymes causing genetic instability and leads to a subsequent block in myeloid differentiation (60). Intracellular (R)-2-HG also inhibits Jumonji-C histone demethylases, resulting increased DNA and histone methylation (61), which reprograms the transcriptional state driving the survival of drug-resistant AML cells (62).

By lowering (R)-2-HG content, *IDH*-mutant specific inhibitors (IDHi) can induce the differentiation of AML cells, yet they have limited clinical efficacy as a monotherapy (63). When combined with agents that inhibit epigenetic DNA modification such as histone methylation, IDHi exhibits a synergistic effect on AML neoplasm. The first-in-class oral drug Enasidenib (AG-221/CC-90007) is a selective inhibitor of *IDH2* mutant enzymes. The Phase 1/2 study showed that median OS in R/R AML patients was 9.3 months. For the 34 (19.3%) patients with complete remission (CR), OS was 19.7 months (4). IDHi combinations with hypomethylating agents may confer additional clinical benefits over either of these therapies alone. In an open-label, Phase 1b/2 clinical trial at 43 sites in 12 countries (AG221-AML-005), 50 (74%) patients in the combination group of enasidenib plus azacitidine achieved an OR rate higher than that in 12 (36%) patients of the azacitidine monotherapy group, suggesting the improved outcome by this combination for the newly diagnosed AML patients with mutant *IDH2* (64). It was recently reported that a 60-year-old patient with refractory AML, with *IDH2* mutations, achieved CR in response to the triple combination of enasidenib, azacytidine, and BCL-2 inhibitor venetoclax (65). A trial examining IDHi in combination with chemotherapy for newly diagnosed AML or myelodysplastic syndrome EB2 with an *IDH1* or *IDH2* mutation is currently ongoing (https://clinicaltrials.gov/, NCT03839771).

### 3.2 CD39

Wildly expressed on vascular endothelial cells, immune cells and tumor cells, CD39 (ecto-nucleoside triphosphate diphosphohydrolase-1) is the dominant and rate-limiting ectonucleotidase that hydrolyzes ATP and adenosine diphosphate (ADP) to adenosine monophosphate (AMP) (66). Under normal physiological conditions, the metabolic homeostasis of the purine pool is maintained through the salvage pathway to reuse the released purine nucleobases in the form of adenine, guanine, and the hypoxanthine base of inosine monophosphate during the breakdown process of nucleic acids (67). In the absence of CD39, with the limitation of free purine nucleobases, the salvage pathway will need to shift to the *de novo* synthesis pathway. Besides the higher energy cost, this metabolic shift also pays a higher price given the increased consumption in amino acids (i.e. glutamine, glycine, aspartate) and additional metabolites (i.e. formate and carbon dioxide) when compared to the salvage pathway (35). In addition to its role in purine metabolism, our colleagues and we have used genetic, pharmacological, and immunological methods to demonstrate the therapeutical potential of CD39 as a potential “novel immune checkpoint”, as in tumor immunotherapy (68–72).

In murine AML models, we have documented the importance of Cd39 on the engraftment and invasiveness of TIB-49 tumor cells. The acquisition of Cd39 *via* trogocytosis from host BM niche onto otherwise Cd39-non-expressing TIB-49 cells resulted in decreased survival of mice inoculated with cancer cells (73). CD39 is also notably expressed in human AML and likely contributes to chemoresistance (74). In cytarabine-resistant leukemic cells from both AML cell lines and patient samples, CD39 expression is upregulated. Compared with that in newly diagnosed patients, CD39 activity also increases in AML patients on chemotherapy. The intrinsic chemoresistance of AML blasts is at least partly contributed by CD39-mediated activation of the P2RY13-cAMP-PKA pathway and the ATF4 axis, triggering mitochondrial ROS production and OXPHOS activity that leads to chemoresistance with heightened antioxidant defenses by AML cells (74).

Thus, targeting CD39 offers a new promising therapeutic strategy to restore metabolic vulnerability in drug-resistant AML cells, at least in part by selectively dampening OXPHOS. Pharmacologic inhibition of CD39 ectonucleotidase activity by POM-1 suppresses mitochondrial reprogramming and enhances the sensitivity of AML blasts to cytarabine (74). Monoclonal antibodies (mAb) neutralizing CD39 ectonucleotidase activity, such as ES002023 (Elpiscience Biopharma, Ltd.), SRF617 (Surface Oncology) and TTX-030 (Tizona Therapeutics), have been developed to boost extracellular ATP anti-tumor responses and curb the immunosuppressive effects of adenosine in tumors. Monotherapies with anti-CD39 mAbs or in combination with other immunotherapy and/or chemotherapy are in the early stage of clinical trials in advanced and metastatic solid tumors (https://clinicaltrials.gov/, NCT05075564, NCT04336098, NCT04306900 and NCT05177770). The role of these anti-CD39 mAbs in AML has not been tested and elucidated.

Instead of blocking CD39 ectonucleotidase activity, our colleagues and we have glyco-engineered anti-mCd39 antibodies (αCd39 mAb) to promote Fc receptor interactions. This approach thereby augments antibody-dependent cellular cytotoxicity and promotes trogocytosis. These mAbs promote anti-cancer responses by depleting Cd39-positive myeloid suppressor cells and inhibiting angiogenesis in murine colon cancer models (75). The anti-hCD39 antibodies, PUR001 (Purinomia Biotech), exhibit similar anti-tumor mechanisms *in vivo*, and are in Phase I clinical trial (https://clinicaltrials.gov/, NCT05234853).

To explore the efficacy of such glyco-engineered αCd39 mAbs in AML therapy, we established TIB-49 aggressive AML models in mice. Current data showed that pretreatment with glyco-engineered αCd39 mAb effectively boosted daunorubicin chemotherapy cytotoxicity (73). The effects of αCd39 mAb on AML cell mitochondrial metabolic reprogramming are now under detailed investigation.

### 3.3 NAMPT

Nicotinamide adenine dinucleotide (NAD^+^) is a metabolite that plays a role in maintaining the mitochondrial membrane potential. NAD^+^ is also central to energy metabolism as a coenzyme for redox reactions, carrying electrons from one reaction to another. Moreover, NAD^+^ is an indispensable cofactor for NAD^+^-dependent enzymes in non-redox reactions, such as sirtuins, CD38 and poly(ADP-ribose) polymerases (76). NAMPT is a rate-limiting enzyme in NAD^+^ biogenesis. NAMPT overexpression has been observed in numerous types of cancers, including AML (77, 78). The involvement of NAMPT in multiple key biochemical processes is the foundation of inhibiting cancer cell NAMPT activities as a potential therapeutic strategy for AML treatment. For example, KPT-9274 is a unique p21-activated kinase 4/NAMPT inhibitor that suppresses the conversion of saturated fatty acids to monounsaturated fatty acids, resulting in apoptosis of AML cells (79). In addition, through the depletion of NAD^+^, KPT-9274 also stalls mitochondrial respiration and glycolysis, and induces apoptosis in AML cells regardless of mutation and genomic subtypes (77). Because of the differences in cellular metabolic states, LSCs but not normal HSCs and their progenitor cells are more sensitive to NAMPT inhibition. Therefore, NAMPT inhibition could be a selective therapeutic strategy targeting LSCs in AML (79).

It has been reported that NAMPT inhibition sensitizes leukemia cells for other chemotherapies, and could be a novel strategy to enhance treatment index (80). Combination of etoposide with FK866, an NAMPT-specific inhibitor, causes increased death of leukemia cell lines compared to etoposide alone (80). The group of Craig Jordan found that nicotinamide metabolism is also involved in mediating resistance to venetoclax in LSCs from relapsed AML. These authors investigated the reason for the low response rate of venetoclax-based regimens in R/R AML patients. By comparing the metabolic profiles of LSCs from R/R AML patients vs. untreated patients, they demonstrated that R/R LSCs had a unique metabolic profile which relied on nicotinamide. In R/R LSCs, NAD^+^ drove OXPHOS by activating the NAD^+^-dependent amino acid and fatty acid catabolism, and circumvented venetoclax-mediated cytotoxic effects (81). Thus, mechanistically, elevated requirement in nicotinamide metabolism defines a vulnerability point of R/R LSCs that may be targeted to overcome venetoclax/azacitidine resistance (81).

### 3.4 OXPHOS

The heightened dependence of AML cells and LSCs on mitochondrial metabolism renders them more sensitive to inhibition of mitochondrial OXPHOS (82). Silencing the expression of mitochondrial electron transfer flavoprotein (ETF) A and ETFB leads to increased mitochondrial stress and apoptosis in AML cells, but has little to no effect on normal human CD34^+^ HSC cells (83). Targeting ETC complexes in OXPHOS has emerged as another attractive anti-leukemia strategy.

When treated with intensive induction chemotherapy, AML patients with strong complex I-dependent respiration and high expression of mitochondrial proteins are found to have poor outcome, reduced remission rate and short OS (84). Besides high TCA cycle intermediates, increased ETC complex I activity is also found to be one of the mechanisms responsible for the enhanced mitochondrial oxidative metabolism in AML patients harboring IDH mutation. OXPHOS inhibitors show synergistic anti-AML efficacy when combined with IDHi *in vivo* (85). Mubritinib, a known inhibitor of ERBB2 (Erb-B2 receptor tyrosine kinase 2), has been reported to elicit strong anti-leukemic effects *in vitro* and *in vivo* through inhibition of ETC complex I activity (86). IACS-010759, a highly selective small-molecule inhibitor of Complex I demonstrates effective inhibition on cell respiration with potent anti-leukemia effect in pre-clinical AML models (87). The combination of IACS-010759 with venetoclax shows synergistic effects in inducing AML cell death (88). A novel synergistic effect between IACS-010759 and FLT3 inhibitor AC220 (quizartinib) is also observed in AML cells. This is likely due to a major disruption of cell metabolism, independent of FLT3 mutation status (89). IACS-010759, however, is found to have a narrow therapeutic window with emergent dose-limiting toxicities in two phase I trials. Also, even at the tolerated doses only modest target inhibition and limited antitumor activity were observed (90). Clearly, further drug studies are needed to expand the dose window and limit normal tissue toxicity before advancing the translation of these compounds.

In this sense, research has been extended to inhibitors of Complex II and Complex III that also display anti-leukemia effects in AML. High activity of mitochondrial ETC Complex II is found in AML patients with FLT3-ITD mutations. Inhibition of ETC Complex II enhances apoptosis in FLT3-ITD^+^ AML cells (91). Genetic knockdown of the ETC Complex II chaperone protein SDHAF1 (succinate dehydrogenase assembly factor 1) delayed AML cell growth *in vitro* and *in vivo*. Moreover, Complex II inhibition induces selective death of AML cells while sparing normal HSCs (92). Pharmacological inhibition of the mitochondrial ETC Complex III by antimycin A is also found to inhibit proliferation and promote cellular differentiation of AML cells (93).

In pediatric AML, the activity of both mitochondrial Complex II and V are significantly elevated in BM mononuclear cells compared to controls (94). In a scientific report studying the mtDNA mutational patterns of pediatric leukemic cases from an endogamous tribal population in Northeast India, non-synonymous variants in mitochondrial Complex V are found to be the driving factors for diseases, demonstrating the role of Complex V in pediatric leukemia development (95).

## 4 Mitochondria trafficking from MSCs to AML and addressing related therapies to overcome chemoresistance in AML

**Figure 3:**
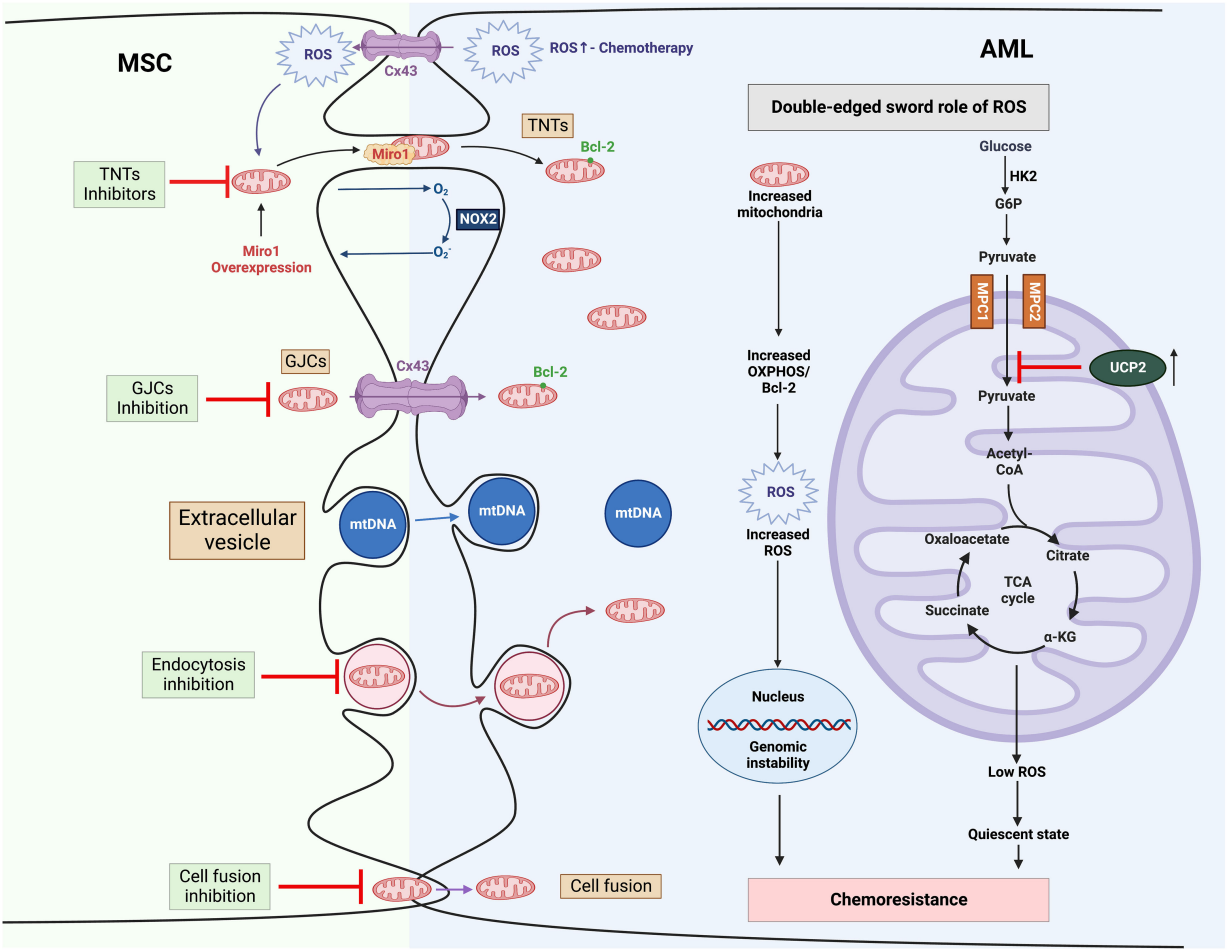
Mitochondria trafficking from MSCs to AML and related therapies to overcome chemoresistance (1). MSCs transfer their mitochondrial cargo to AML cells *via* various mechanisms, including TNTs, GJCs, extracellular vesicles, and cell fusion (2). Mitochondria trafficking can be further boosted by chemotherapy. Increased mitochondria contribute to high ROS levels in AML, which in turn promotes mitochondria trafficking from MSCs as a positive feedback mechanism (3). In AML, high ROS drives genomic instability, leading to chemotherapy resistance. MSCs stimulate the expression of UCP2 in leukemic cells, which suppresses the import of pyruvate into mitochondria, inducing a “Warburg phenotype”, and reduces the production of ROS, engendering AML cells to be in a quiescent state and resistant to chemotherapy. α-KG, α-ketoglutarate; AML, Acute myeloid leukemia; BCL-2, B-cell lymphoma 2; Cx43, Connexin 43; G6P, glucose 6-phosphate; GJCs, gap-junction channels; HK2, hexokinase 2; MPC, mitochondrial pyruvate carrier; MSC, mesenchymal stromal cell; mtDNA, mitochondrial DNA; NOX2, nicotinamide adenine dinucleotide phosphate oxidases 2; OXPHOS, oxidative phosphorylation; ROS, reactive oxygen species; TCA cycle, tricarboxylic acid cycle; TNTs, tunnelling nanotubes; UCP2, uncoupling protein 2.

Multiple mechanisms have been proposed, whereby MSCs, essential components in BM niche, appear to protect leukemic cells from multiple therapies. These properties include secreting pro-survival factors (e.g., cytokines, chemokines growth factors), providing metabolic substrates as an alternative to glucose (e.g., amino acids and fatty acids) and rewiring metabolic programs (96). Here, we will focus on MSC-related mitochondria metabolic reprogramming in AML (**Figure 3**).

### 4.1 Mitochondria trafficking from MSCs

MSCs have been documented to transfer their mitochondria to AML cells in the BM microenvironment (41). One way to quickly rescue and reboot the biological functions of stressed recipient cells is horizontal transfer of mitochondria or mitochondrial genomes from healthy donor cells, in this case, BM MSCs. MSCs are also believed to protect cancerous cells by regulation of cellular metabolism during chemotherapies (97). Blocking stromal cells to AML cells mitochondrial transfer in BM niche was found to sensitize AML cells to chemotherapy (33). Besides mitochondrial transfer, MSCs stimulate uncoupling protein 2 (UCP2) expression in leukemia cells and induce AML cells chemoresistance (98). Pyruvate is a key metabolite in mitochondria metabolism as it is at the crossroads of mitochondrial OXPHOS and cytoplasmic glycolysis. Pyruvate “import” into mitochondria is suppressed by high expression of UCP2, inducing a “Warburg phenotype” in AML cells. The uncoupling of mitochondria and associated low ROS levels engender cellular metabolic states, phenotypically similar to LSCs. These factors may contribute to the resistance to conventional chemotherapy. Targeting MSCs may be a novel therapeutic avenue to overcome chemoresistance.

Cells transfer their mitochondrial cargo to other cells *via* various mechanisms. One mechanism is mediated by tunneling nanotubes (TNTs), which help cells to “intercommunicate” directly with each other across lengthier spaces (99). A wide variety of cellular components of diverse sizes, such as mitochondria, exosomes, microvesicles, lysosomes, proteins, microRNAs and even ions can be transferred through TNTs (100). Another mechanism for mitochondria transport involves gap-junction channels (GJCs), formed by Connexin (Cx) 43 (Cx43) hemichannels (101). Through connecting two apposed hexameric Cx hemichannels, Cx proteins establish GJCs to enable direct exchange of cellular components between cells or with the extracellular milieu (102). In addition to these cellular “bridges”, MSCs can also transfer to recipient cells mtDNA and mitochondrial per se through secretion of extracellular vesicles (103). Cell fusion is yet another mechanism used by MSCs for mitochondria exchange, in which uninuclear cells merge plasma membranes into multinuclear cells and thus share organelles and cytosolic components (104). Mitochondria trafficking can be further boosted by chemotherapeutics routinely used in clinic, such as cytarabine, etoposide and doxorubicin, and is associated with increased OXPHOS-derived ATP production (41). AML cells gain survival advantages and resistance to standard therapy when equipped with transferred mitochondria as well as other anti-apoptosis proteins, e.g., BCL-2 family proteins (105).

Therefore, targeting “mitochondria smuggling” from MSCs into AML cells forms the basis for novel therapeutic approaches (mitotherapies) against chemoresistance empowered by donor mitochondria. As normal CD34^+^ HSCs are less likely to receive extra mitochondria, this mitotherapy could have a good therapeutic window with less side effects (41). Curiously, the surface molecule CD38 appears to play a critical role in mitochondria transport from MSCs to AML blasts in the BM microenvironment (106). Daratumumab, an anti-CD38 mAb approved for the treatment of multiple myeloma, has been shown to inhibit this process (107). Targeting NAD^+^-dependent CD38 may have an additional benefit intrinsically rooted in the link of NAD biogenesis in mitochondria. Although many chemotherapeutic agents and conventional anti-cancer drugs have been identified to reduce TNT formation, such as cytarabine, daunorubicin, everolimus, cytochalasin D, latrunculin A and B, metformin, nocodazole CK-666, ML-141, 6-thio-GTP, BAY-117082, and octanol (108), they nonetheless could trigger negative effects on basic cellular functions as the actin constituent of TNTs is also part of the cytoskeleton (99). Miro1 is a Ca²^+^-sensing adaptor protein that tethers mitochondria to the trafficking apparatus. This tethering is abrogated by micromolar levels of Ca^2+^ binding to Miro1 (109). Miro1 overexpression in MSCs enhances the transfer of mitochondria while Miro1 depletion inhibits mitochondria transfer (110). Disruption of GJCs with carbenoxolone (CBX) attenuates AML chemoresistance induced by MSCs and synergizes with cytarabine *in vitro* and *in vivo*. CBX’s proapoptotic effect on AML cells is in line with the extinction of energy metabolism (111). Targeting endocytosis mediated by NAD^+^-CD38-cADPR-Ca^2+^ signaling could also be a promising approach to block mitochondrial transfer (112).

### 4.2 ROS in AML

ROS-mediated “redox signaling” controls basic cellular functions by modifying the activity and expression of various metabolic enzymes and transcription factors (113). An important primary source of ROS in leukemia is generated by NADPH oxidases (NOX). Over 60% of primary AML blasts are found to synthesize high levels of ROS by NOX, promoting AML cell proliferation and survival. Another major source of endogenous ROS comes from the ETC complex (114). Mitochondria transfer from MSCs to AML cells is associated with high ROS levels in AML, which in turn increase mitochondrial uptake from MSCs as a positive feedback mechanism (41, 115). The mutant receptor kinases *FLT3*, *IDH1/2*, *cKIT* and *RAS* also seem to drive ROS production in AML cells (116). What’s more, many antioxidant systems seem to be defective in AML, leading to increased overall ROS levels (114, 117, 118). However, the intracellular ROS levels differ in AML cell subpopulations. While bulk AML cells are characterized by high ROS levels, AML LSCs exhibit low cellular oxidative status, which results from a combination of low mitochondrial activity and high capacity of ROS removal (51). In LSCs, a low ROS level is related to their quiescent state and resistance to the conventional chemotherapy.

Altered ROS homeostasis has detrimental effects as a consequence of damage to protein, lipid and DNA; latter leading to subsequent cell apoptosis, new mutations and leukemic clones (117). Through activation of kinases and inactivation of protein tyrosine phosphatases, high-level ROS production drives second messenger signaling, increased *FLT3* signaling, lipid peroxidation and genomic instability, leading to chemotherapy resistance (116). High levels of ROS are also suggested to drive leukemogenesis (119).

New therapeutic approaches have been developed to potentially rectify the imbalance of cellular ROS (116, 120). Antioxidants are used to counteract the deleterious effects of high ROS in AML. The natural compound vitamin C (ascorbic acid) has emerged as a potential anti-proliferative and pro-apoptotic agent on leukemic cells through epigenetic regulations and scavenge of ROS to prevent DNA damage (121). Effectiveness remains controversial as antioxidants may limit the effectiveness of chemotherapy by protecting malignant cells. Studies also confirm that decreasing ROS levels may be a double-edged sword in AML treatment. When applied as a single agent, decitabine induces the activation of NRF2 and downstream antioxidative response. This restrains ROS generation, leading to decitabine resistance. Adding all-trans retinoic acid blocks NRF2 activation, resulting in ROS accumulation and ROS-dependent cytotoxicity (122). A similar phenomenon is also seen when AML patients are treated with arsenic trioxide and venetoclax, which cooperatively induce LSC apoptosis through potentiation of ROS induction (123). Setanaxib (GKT137831), a clinically advanced ROS-modulating agent, shows antiproliferative activity and potently enhances the cytotoxic action of anthracyclines *in vitro* through enhancing anthracycline-induced ROS formation in AML cells (124). Furthermore, low levels of ROS in AML will facilitate entry of AML cells to the quiescent state and to become resistant to the conventional chemotherapy (51). Therefore, close regulation of levels of ROS, as in settings of AML treatment, need to be carefully studied.

## 5 Conclusion and future perspective

The growing quest for novel therapeutic strategies together with continuous elucidation of metabolic pathophysiology in AML might help explain how blasts and LSCs develop resistance to current therapeutic regimens. Resistance to chemotherapy appears secondary to both intrinsic cellular factors and extrinsic environmental events. Metabolic reprogramming is one of those intrinsic factors that helps to protect AML viability and furthermore induces resistance to therapeutic agents. The interactions between MSCs and AML cells, especially involving mitochondria transfer from MSCs to AML blasts and LSCs, are also extrinsic niche-specific events. These pathways possibly further facilitate chemoresistance.

The successful application of mitotherapy in leukemia will require normalization of aberrant cellular pathophysiology processes (e.g., disordered mitochondria metabolism) within blast cells. Therapeutic strategies that address mitochondria biology and metabolic reprogramming (mitotherapies) may provide more specific and potent regimens for AML, particularly when used in combination with chemotherapy and potentially also immunotherapy.

## Author contributions

Conceptualization, LF, JY, and SR; writing – original draft preparation, LF and PZ; writing – review and editing, WG, JY, and SR; visualization, LF; supervision, JY and SR; funding acquisition, SR. All authors contributed to the article and approved the submitted version.
